# *N*-(4-Methoxyphenyl)Pentanamide, a Simplified Derivative of Albendazole, Displays Anthelmintic Properties against the Nematode Toxocara canis

**DOI:** 10.1128/spectrum.01807-22

**Published:** 2022-07-28

**Authors:** Taís C. Silva, Ana C. Mengarda, Bruna L. Lemes, Susana A. Z. Lescano, Dalete Christine S. Souza, João Henrique G. Lago, Josué de Moraes

**Affiliations:** a Núcleo de Pesquisa em Doenças Negligenciadas, Universidade Guarulhos, Guarulhos, São Paulo, Brazil; b Instituto de Medicina Tropical, Faculdade de Medicina, Universidade de São Paulo, São Paulo, São Paulo, Brazil; c Centro de Ciências Naturais e Humanas, Universidade Federal do ABC, Santo André, São Paulo, Brazil; Weill Cornell Medicine

**Keywords:** neglected diseases, antiparasitic agents, molecular simplification, albendazole, *Toxocara canis*, toxocariasis, helminthiasis

## Abstract

Infections caused by parasitic helminths have enormous health, social, and economic impacts worldwide. The treatment and control of these diseases have been dependent on a limited set of drugs, many of which have become less effective, necessitating the search for novel anthelmintic agents. In this study, a simplified compound, *N*-(4-methoxyphenyl)pentanamide (N4MP), based on the structure of the most widely used anthelmintic (albendazole), was chemically prepared using 4-anisidine and pentanoic acid. *N*-(4-Methoxyphenyl)pentanamide was evaluated *in vitro* against the nematode Toxocara canis, an ascarid roundworm of animals that can infect humans. Similar to albendazole, bioassays showed that *N*-(4-methoxyphenyl)pentanamide affected the viability of parasites in a time- and concentration-dependent manner. Interestingly, *N*-(4-methoxyphenyl)pentanamide showed a profile of lower cytotoxicity to human and animal cell lines than albendazole. Pharmacokinetic, drug-likeness, and medicinal chemistry friendliness studies demonstrated an excellent drug-likeness profile for *N*-(4-methoxyphenyl)pentanamide as well as an adherence to major pharmaceutical companies’ filters. Collectively, the results of this study demonstrate that the molecular simplification of albendazole to give *N*-(4-methoxyphenyl)pentanamide may be an important pipeline in the discovery of novel anthelmintic agents.

**IMPORTANCE** Infections caused by parasitic helminths have enormous health, social, and economic impacts worldwide. The treatment and control of these diseases have been dependent on a limited set of drugs, many of which have become less effective, necessitating the search for novel anthelmintic agents. Considering this scenario, the present study reports the preparation of *N*-(4-methoxyphenyl)pentanamide (N4MP), a simplified molecule based on the structure of the most widely used anthelmintic (albendazole). N4MP was evaluated *in vitro* against the nematode Toxocara canis, a common ascarid roundworm of domestic animals that can infect humans. Similar to albendazole, bioassays showed that N4MP affected the viability of parasites in a time- and concentration-dependent manner but displayed a profile of lower cytotoxicity to human and animal cell lines than albendazole. Therefore, this study demonstrates that the molecular simplification of albendazole to give N4MP may be an important pipeline in the discovery of novel anthelmintic agents.

## INTRODUCTION

Infections caused by parasitic helminths have enormous health, social, and economic impacts worldwide and disproportionally affect the poorest and most deprived communities (1). These diseases are also of enormous animal health significance, with economic and social impacts in both developed and developing countries (2). Soil-transmitted helminthiasis (STH) infections are among the most common infections worldwide, and these worms produce a wide range of symptoms, including intestinal manifestations (e.g., abdominal pain and diarrhea), general malaise, and weakness. In addition, STH infections are associated with malnutrition, impaired growth, and poor school performance (3).

Globally, approximately 25% of the world’s population is infected with STH (3), with Ascaris lumbricoides alone infecting over 800 million people (4). Given the critical role that these conditions play in global health, in 2021, the World Health Organization (WHO) launched a new roadmap for neglected diseases for 2021 to 2030 (5). Aiming to eliminate STH and other helminthiases as a public health problem, the strategy for the control of infections is to control morbidity through the periodic treatment (once or twice per year) of at-risk people living in areas of endemicity. The WHO roadmap also advocates integrated approaches for the treatment of disease in animals and humans. However, there are only a few recommended anthelmintics available for the treatment and control of helminth infections, with albendazole currently being the most widely used drug. There is a pressing need for new drugs to fight helminthiasis, and efforts are under way to identify novel chemical entities with anthelmintic properties (6).

Among the many ascarid nematodes that exist in nature, *Ascaris lumbricoides* is the main species that infects people, whereas Toxocara species are the most important ascarids in domestic animals. Of the different Toxocara species, T. canis and T. cati are considered to pose the highest zoonotic risk. As a neglected disease, human toxocariasis afflicts millions of children and adolescents, particularly in impoverished communities around the world. Although albendazole is the first choice for the treatment of toxocariasis, it has limited efficacy against tissue larvae (7). In addition, albendazole has very low intestinal absorption and undergoes extensive first-pass metabolism (8). Furthermore, albendazole has been used for many decades, and several hundred million doses are used in areas of endemicity for STH control (9). Hence, its consistent use makes it prone to drug resistance, a phenomenon common in veterinary medicine (10).

Since reducing the burden of parasitic diseases that affect people in the developing world requires sustained collaborative drug discovery efforts (6), in this study, a simplified compound, *N*-(4-methoxyphenyl)pentanamide (N4MP), based on the structure of albendazole, was chemically prepared, and its activity was evaluated *in vitro* against infective third-stage larvae (L3) of *T. canis.* In addition, analyses of physicochemical properties, pharmacokinetics, drug-likeness, and medicinal chemistry friendliness were performed with albendazole and the simplified derivative *N-*(4-methoxyphenyl)pentanamide.

## RESULTS AND DISCUSSION

### Molecular simplification of albendazole.

**FIG 1:**
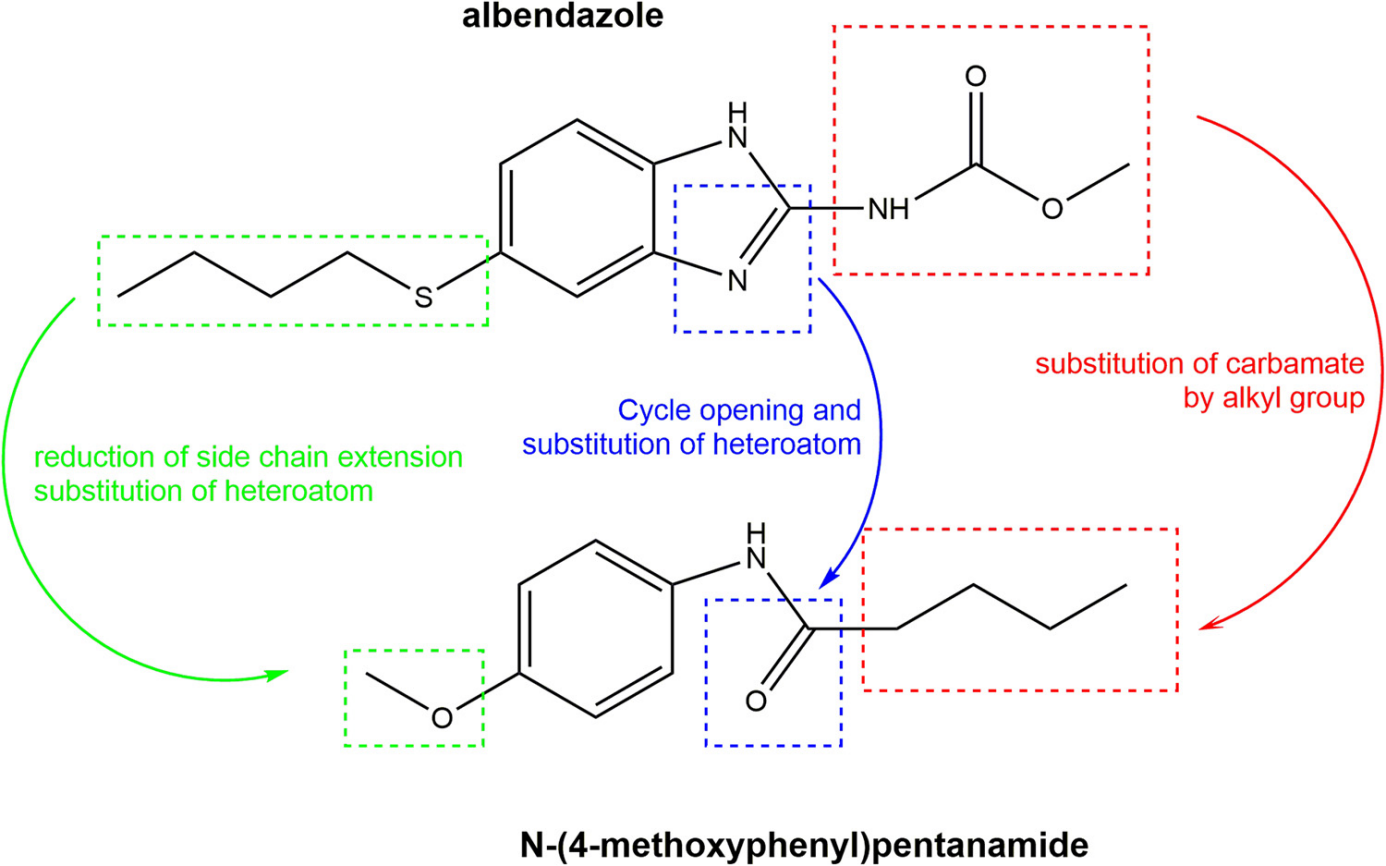
Molecular simplification of albendazole to give *N*-(4-methoxyphenyl)pentanamide. Main chemical simplifications were performed in the C-4 position of the aromatic ring (green), the guanidine unity (blue), and the carbamate moiety (red).

Initially, we planned to prepare a simplified derivative of albendazole, including the reduction of the side chain, the replacement of sulfur by an oxygen atom at the C-4 position, the replacement of guanidine function by an amide group, and the replacement of the carbamate group by a similar alkyl chain, resulting in *N*-(4-methoxyphenyl)pentanamide, as indicated in Fig. 1. This compound was thus prepared by using 4-anisidine and pentanoic acid and chemically characterized by analyses of nuclear magnetic resonance (NMR) and electrospray ionization–high-resolution mass spectrometry (ESI-HRMS) spectra (see Fig. S1 to S4 at https://npdn.com.br/material-suplementar).

### Physicochemical properties and drug-likeness parameters of albendazole and its simplified derivative.

**FIG 2:**
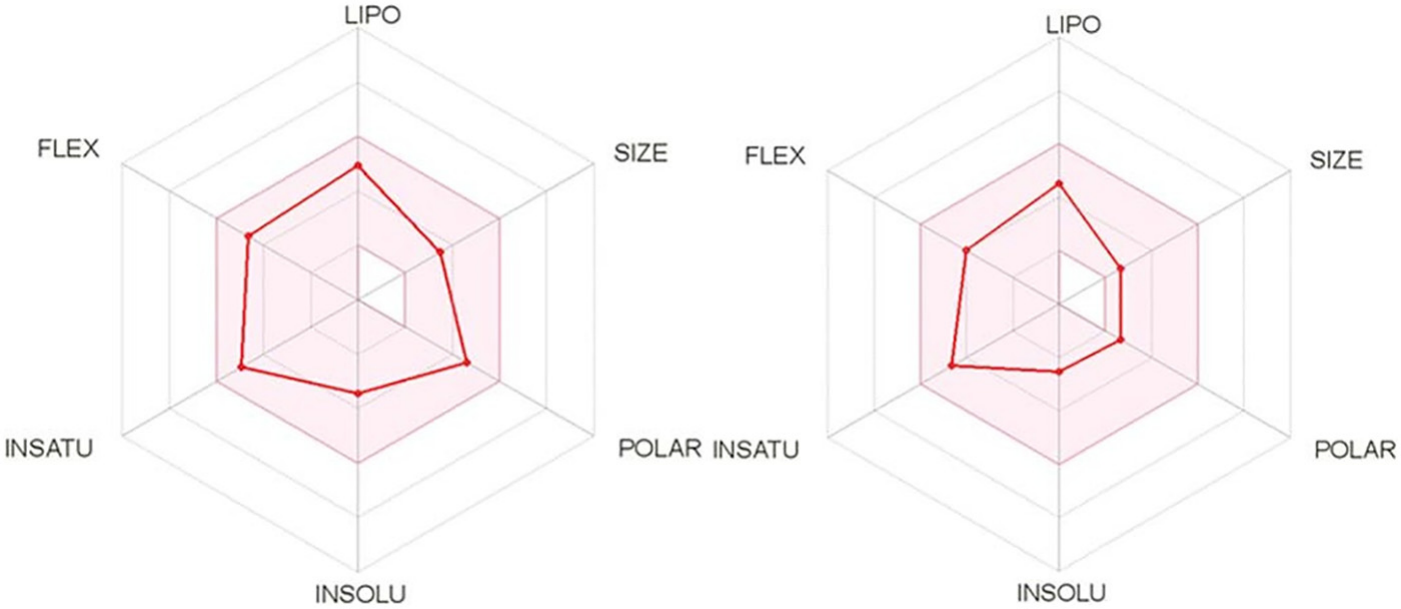
*In silico* study of albendazole (left) drug-likeness for *N*-(4-methoxyphenyl)pentanamide (right) using the SwissADME platform.

As an initial *in silico* evaluation, different parameters such as polarity (POLAR), molecular weight (SIZE), flexibility (FLEX), lipophilicity (LIPO), insolubility (INSOLU), and unsaturation (INSATU) were determined using the SwissADME platform for albendazole and the simplified compound *N*-(4-methoxyphenyl)pentanamide. As indicated in the bioavailability radar (Fig. 2), the tested compounds demonstrated excellent adherence to all evaluated parameters.

*In silico* physicochemical, pharmacokinetic, and drug-likeness predictions for albendazole and its simplified derivative *N*-(4-methoxyphenyl)pentanamide (Table 1) resulted in excellent adherence to “BigPharma” filters such as the Ghose, Veber, Egan, and Muegge filters, including no violations of Lipinski’s rule of five (RO5). These data suggested adequate drug ability of the simplified derivative *N*-(4-methoxyphenyl)pentanamide, with topological polar surface area (TPSA) values in accordance with those reported in the literature for lead compounds, adequate gastrointestinal (GI) absorption and log *P* values, as well as high solubility in water, supporting its use as an oral candidate compound.

**TABLE 1:** Physicochemical, pharmacokinetic, and drug-likeness predictions for albendazole and the simplified compound *N*-(4-methoxyphenyl)pentanamide^*a*^

Parameter	Value for compound	
	Albendazole	*N*-(4-Methoxyphenyl)pentanamide
Log *P*	2.63	2.45
No. of HBA groups	3	2
No. of HBD groups	2	1
TPSA (Å^2^)	92.31	38.33
Solubility	Soluble	Soluble
GI absorption level	High	High
BBB permeant	No	Yes
CYP1A2 inhibitor	Yes	Yes
Adherence to filter		
Lipinski	Yes	Yes
Ghose	Yes	Yes
Veber	Yes	Yes
Egan	Yes	Yes
Muegge	Yes	Yes
PAINS	0 alert	0 alert
Synthetic accessibility	2.58	1.34

a Log *P*, logarithm of *n*-octanol–water; HBD, hydrogen bond donor; HBA, hydrogen bond acceptor; TPSA, topological polar surface area; GI, gastrointestinal; BBB, blood-brain barrier; CYP1A2, cytochrome P450 family 1 subfamily A member 2, involved in the metabolism of xenobiotics; PAINS, panassay interference substructures.

Furthermore, it was possible to predict that *N*-(4-methoxyphenyl)pentanamide is able to permeate the blood-brain barrier (BBB), an important aspect that selectively regulates the permeation of drugs into the brain. As shown in Table 1, both of the tested compounds were predicted to be CYP inhibitors, an important aspect associated with the metabolism of xenobiotics.

Additionally, the applied filters used suggested that both compounds could not be considered panassay interference substructures (PAINS), caused by possible promiscuous reactivity. Finally, synthetic accessibility, an important aspect associated with the development of new drugs for neglected diseases, was calculated to be 2.58 for albendazole, approximately 2-fold higher than the value calculated for *N*-(4-methoxyphenyl)pentanamide (1.34), indicating ease in the synthesis of this compound in order to corroborate the simplification approach.

### Anthelmintic activity against *T. canis*.

**FIG 3:**
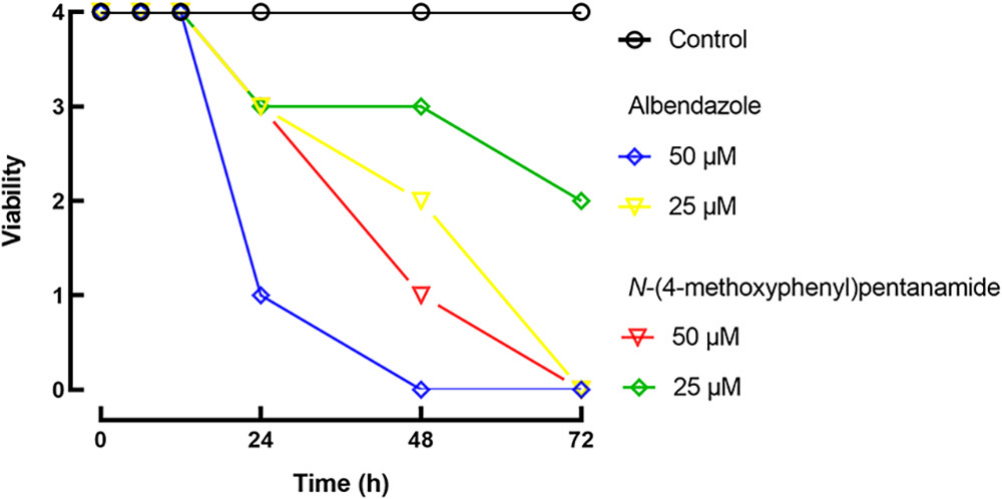
Concentration- and time-dependent antiparasitic activity of anthelmintic drugs against *T. canis* L3. Parasites were exposed to compounds, and viability was scored at indicated time points as 0 (dead), 1 (immotile), 2 (intermittent shaking of the head or tail region), 3 (sluggish and motile), or 4 (highly active and motile). Data points represent results from at least three independent experiments conducted in triplicate (each replicate contains 100 larvae).

The larval motility assay is currently the method of choice to evaluate the drug sensitivities of different nematode species (11, 12). Thus, assays to determine the viability of infective third-stage larvae (L3) of *T. canis* were performed with albendazole and the simplified compound *N*-(4-methoxyphenyl)pentanamide to determine the concentration- and time-dependent effects. The viability of *T. canis* L3 over a period of 72 h *in vitro* is demonstrated in Fig. 3.

Control worms remained viable over the entire observation period. Albendazole and its simplified compound were able to kill all *T. canis* L3 within 72 h of contact at a concentration of 50 μM. In the temporal analysis, *T. canis* larvae exposed to either of the two compounds seemed to suffer no apparent decrease in their motility in the early hours (6 and 12 h), consistent with observations reported previously where *T. canis* larvae do not die immediately after exposure to flubendazole (13). Comparatively, a slightly slower onset of action was observed when parasites were exposed to *N*-(4-methoxyphenyl)pentanamide after 24 h. In more detail, at a concentration of 50 μM, L3 were immobilized when exposed to albendazole and *N*-(4-methoxyphenyl)pentanamide after 24 and 48 h, respectively. At the same concentration, the death of all parasites exposed to albendazole and *N*-(4-methoxyphenyl)pentanamide was observed after 48 and 72 h, respectively. It should be noted that the reduction and change in motility should be sufficient *in vivo* to expel the worms from the host’s intestine, as has been shown previously for other anthelmintics (14).

The mechanism by which *N*-(4-methoxyphenyl)pentanamide is active against *T. canis* has not been explored. However, in roundworms, albendazole and other benzimidazoles bind to tubulins of susceptible worms, preventing their incorporation into microtubes. As cytoplasmic microtubules are critical for promoting glucose uptake, the glycogen stores of the parasites are depleted, leading to the death of the parasites (15, 16). Interestingly, *N*-(4-methoxyphenyl)pentanamide demonstrated anthelmintic activity similar to that of albendazole, and hence, this study may provide the basis for the future development of new albendazole preparations.

### Cytotoxicity evaluation.

**FIG 4:**
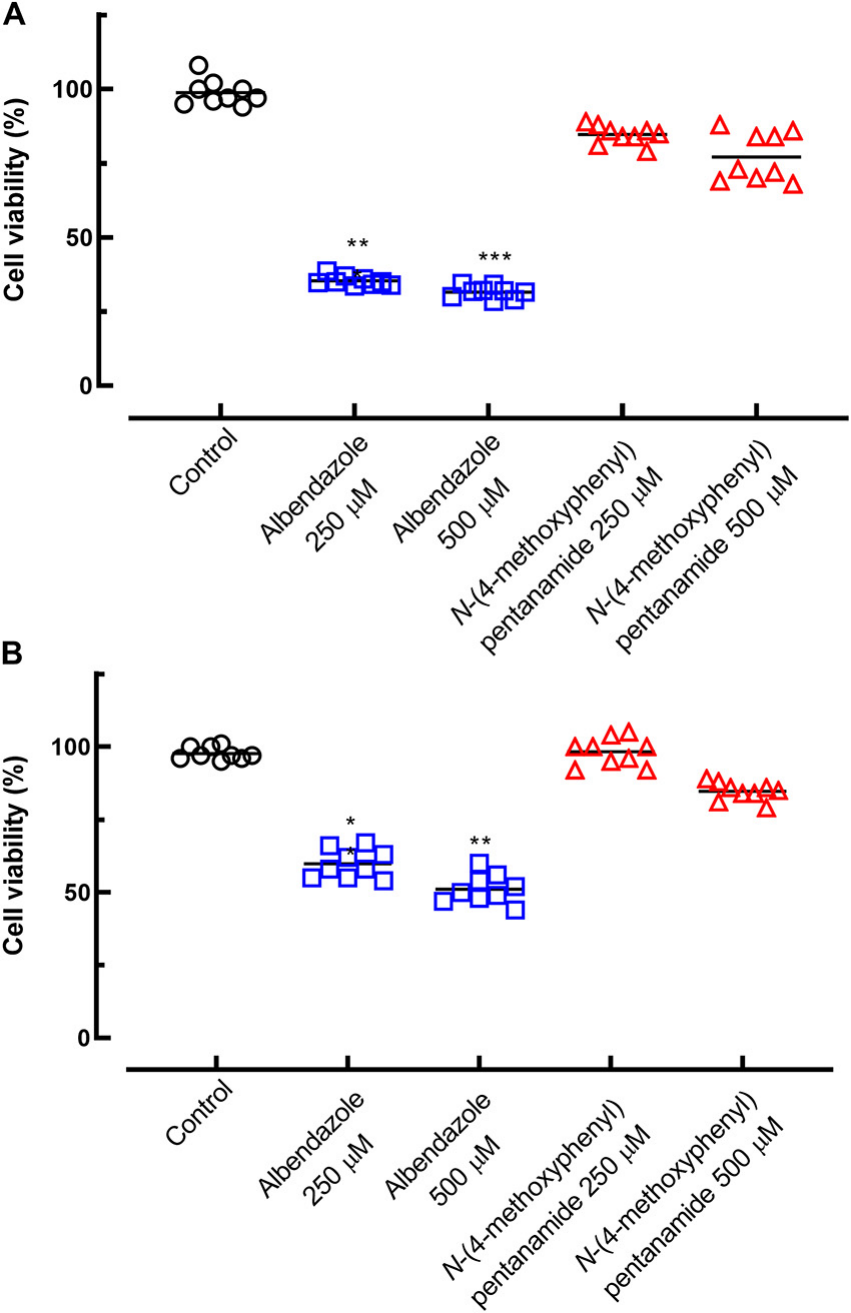
Assessment of the profiles of toxicity of albendazole and *N*-(4-methoxyphenyl)pentanamide toward human (A) and animal (B) cells. The cells were treated with compounds, and viability was estimated by an MTT assay. Points represent data from individual wells from three independent experiments performed in triplicate. **, *P < *0.01; ***, *P < *0.001 (versus the control).

The next step was to estimate the toxicity of albendazole and the simplified derivative *N*-(4-methoxyphenyl)pentanamide by testing on a human cell line (SH-SY5Y) and a monkey cell line (Vero). Cells were incubated with each compound at 250 and 500 μM, i.e., at least 10 times higher than the antiparasitic activity, to achieve the recommended minimum selectivity index (SI) (SI ≥ 10) (17). As shown in Fig. 4, albendazole showed a significant cytotoxicity profile on both human and animal cells, with reductions in cell viability of approximately 30% (*P < *0.001) and 50% (*P < *0.01), respectively. In the literature, albendazole and many benzimidazole derivatives were reported to be cytotoxic to various cell lines (18–20), in line with the results of the present study. Comparatively, *N*-(4-methoxyphenyl)pentanamide had a profile of significantly reduced cytotoxicity relative to albendazole. Thus, *N*-(4-methoxyphenyl)pentanamide showed significant antiparasitic activity against *T. canis*, without any toxicity toward human and animal cells. This remarkable selective antiparasitic effect of *N*-(4-methoxyphenyl)pentanamide provokes interest, in the future, in the synthesis of novel albendazole derivatives as medicinal scaffolds against Toxocara.

### Conclusion.

The development of novel drugs with minimal intrinsic toxicities and low manufacturing prices remains highly desirable to strengthen the pharmaceutical arsenal against helminthiasis. In this study, *N*-(4-methoxyphenyl)pentanamide demonstrated anthelmintic activity similar to that of albendazole. More importantly, *N*-(4-methoxyphenyl)pentanamide was less toxic than albendazole. In addition, *in silico* pharmacokinetic, drug-likeness, and medicinal chemistry friendliness studies demonstrated an excellent drug-likeness profile for *N*-(4-methoxyphenyl)pentanamide as well as adherence to major pharmaceutical company filters. Thus, collectively, the results of this study provide experimental guidance for the future development of new and more selective simplified albendazole derivatives.

## MATERIALS AND METHODS

### General procedures.

All laboratory reagents were of analytical grade (Sigma-Aldrich). Silica gel (230 to 400 mesh; Merck) was used for column chromatographic separation, while silica gel 60 PF_254_ (Merck) was used for thin-layer chromatography (TLC). NMR spectra were recorded on a Varian Inova 500 spectrometer operating at 500 and 125 MHz for ^1^H and ^13^C nuclei, respectively, and equipped with a 5-mm probe, using CDCl_3_ (Aldrich) as a solvent and as an internal standard. Chemical shifts are reported in δ units (parts per million), and coupling constants (*J*) are reported in hertz. The ESI-HRMS spectrum was measured on a Bruker Daltonics MicroTOF QII spectrometer using electrospray ionization in the positive mode.

### Synthesis procedure.

Pentanoic acid (0.4 g; 3.5 mmol) and 4-anisidine (0.45 g; 3.5 mmol) were added to a solution containing 4-dimethylaminopyridine (DMAP) (0.5 g; 4.2 mmol) and 1-(3-dimethylaminopropyl)-3-ethylcarbodiimide hydrochloride (EDCI) (0.75 g; 3.9 mmol) in CH_2_Cl_2_ (10 mL) at room temperature. The reaction mixture was stirred at room temperature for 4 h, diluted with CH_2_Cl_2_ (10 mL), and washed with 5 mL of aqueous HCl (1 mol/L). The organic layer was separated, and the aqueous layer was extracted with CH_2_Cl_2_ (3 times with 10 mL). The combined organic phases were washed with brine, dried over anhydrous Na_2_SO_4_, filtered, and concentrated under reduced pressure. The obtained product was chromatographed over a silica gel eluted with *n*-hexane–ethyl acetate (EtOAc) at 8:2, 7:3, and 1:1 to afford *N*-(4-methoxyphenyl)pentanamide as a white amorphous solid (0.5 g).

For *N*-(4-methoxyphenyl)pentanamide, the yield was 69% and the purity was 99% (high-performance liquid chromatography [HPLC]); ^1^H NMR (500 MHz, CDCl_3_) δ 7.39 (d, *J *= 8.1 Hz, H-2/H-6), 6.84 (d, *J *= 8.1 Hz, H-3/H-5), 3.78 (s, OCH_3_), 2.40 (t, *J *= 7.5 Hz, H-2′), 1.62 (m, H-3′), 1.31 (m, H-5′), 0.90 (t, *J *= 7.3 Hz, H-6′); ^13^C NMR (125 MHz, CDCl_3_) δ 171.7 (C-1′), 156.5 (C-4), 131.3 (C-1), 122.0 (C-2/C-6), 114.2 (C-3/C-5), 55.6 (OCH_3_), 37.5 (C-2′), 27.9 (C-3′), 22.6 (C-4′), 14.0 (C-5′); ESI-HRMS *m/z* 208.1331 [M + H]^+^ (calulated for C_12_H_28_NO_2_, 208.1337).

### *In silico* studies and drug-likeness assessment.

Physicochemical properties and predictions of pharmacokinetic parameters for albendazole and *N*-(4-methoxyphenyl)pentanamide were obtained using the SwissADME Web server (http://www.swissadme.ch/), as indicated in Table 1. Initially, BigPharma filters such as the Ghose, Veber, Egan, Muegge, and Lipinski filters were determined. Other parameters such as log *P* (logarithm of *n*-octanol–water), HBA (hydrogen bond acceptor), HBD (hydrogen bond donor), TPSA (topological polar surface area), and water solubility values were calculated. Other parameters such as values for gastrointestinal (GI) absorption, blood-brain barrier (BBB) penetration, the potential to inhibit CYP1A2 isoforms, the presence of panassay interference substructures (PAINS), and synthetic accessibility were also determined (21, 22).

### Drugs and reagents for antiparasitic assays.

RPMI 1640 culture medium, Dulbecco’s modified Eagle medium (DMEM), antibiotic solutions (10,000 U/mL penicillin G sodium salt and 10 mg/mL streptomycin sulfate), and heat-inactivated fetal bovine serum were purchased from Vitrocell (Campinas, SP, Brazil). HEPES buffer, thiazolyl blue tetrazolium bromide [3-(4,5-dimethyl-2-thiazolyl)-2,5-diphenyl-2H-tetrazolium bromide (MTT)], trypan blue dye, a glutaraldehyde solution, and dimethyl sulfoxide (DMSO) were obtained from Sigma-Aldrich (St. Louis, MO, USA). Albendazole was kindly provided by Ecovet Indústria Veterinária Ltd. (São Paulo, SP, Brazil).

### Parasites.

Toxocara canis adult worms were collected from naturally infected dogs and placed into a solution containing 0.9% sodium chloride. *T. canis* eggs were removed from the uterus of the female worms and incubated in a 2% formalin solution for 30 days at 28°C to induce embryogenesis. *T. canis* third-stage larvae (L3) were obtained as described previously (23) and maintained in RPMI 1640 medium containing 1% (vol/vol) penicillin-streptomycin at 37°C with 5% CO_2_ (24).

### *In vitro* anthelmintic assay.

*In vitro* drug testing was performed as previously reported for other anthelmintic assays (25, 26). Briefly, *T. canis* L3 were transferred to 96-well culture microplates (Corning, New York, NY, USA) containing 100 larvae/well in RPMI 1640 medium. The larvae were treated with the *N*-(4-methoxyphenyl)pentanamide and albendazole (previously dissolved in DMSO) at concentrations of 50, 25, 12.5, 6.24, and 3.1 μM and maintained for 72 h. DMSO was added to the medium of the treated groups and controls in order to obtain a final concentration of 0.2% during the periods of treatment. Each drug concentration was tested in at least triplicate, and the experiments were repeated three times. The viability of larvae was scored immediately after adding the drug (time zero) and after 6, 12, 24, 48, and 72 h using an inverted microscope (INV 100; BEL Engineering, Monza, MB, Italy). Larval motility was scored (effect of ≥60%) as 4 (highly active, using the whole body), 3 (slow motion, using the whole body), 2 (movement with only one part of the body), 1 (immotile but not dead), or 0 (dead). The mortality of *T. canis* larvae was assessed by trypan blue dye exclusion (26).

### Cytotoxicity assay.

The toxic effects of *N*-(4-methoxyphenyl)pentanamide and albendazole were assessed on animal cells (Vero) (African green monkey kidney cells obtained from the American Type Culture Collection [ATCC], Manassas, VA, USA) and human cells (HaCaT) (human keratinocytes obtained from the Banco de Células do Rio de Janeiro [BCRJ], RJ, Brazil) using MTT. Cells were prepared and cultured in DMEM containing 2 mM l-glutamine, a 1% penicillin-streptomycin solution, and 10% fetal bovine serum at 37°C in 5% CO_2_ (Panasonic) as previously described (27, 28). Briefly, cells were seeded into a 96-well plate (Corning) in DMEM with 2-fold serial dilutions of the drug starting at 500 μM for 72 h at 37°C in 5% CO_2_. After the addition of MTT (10 mg/mL; 10 μL per well), the cells were maintained at 37°C for 4 h. The absorbance was estimated at 575 nm by using an Epoch microplate reader (BioTek Instruments, Winooski, VT, USA). The assay was conducted in triplicate and repeated three times. Values were expressed as a percentage of the control.

### Statistical analysis.

All statistical analyses were performed using GraphPad Prism software 8.0. Each assay was performed in triplicate (100 larvae for each replicate, giving a total of 300 larvae for each concentration tested or the control) and repeated at least three times on different days (29). Data are presented as means ± standard deviations (SD).
